# Direct In Situ Conversion of Both Lignin and Hemicellulose into Single Functional Biopolymers via Biomass Fractionation Process

**DOI:** 10.3390/polym17081029

**Published:** 2025-04-10

**Authors:** Caiyun Liu, Shuzhen Ni, Zhaojiang Wang, Yingjuan Fu, Menghua Qin, Yongchao Zhang

**Affiliations:** 1 State Key Laboratory of Green Papermaking and Resource Recycling, Qilu University of Technology, Shandong Academy of Sciences, Jinan 250353, China; 2 School of Chemistry and Chemical Engineering, Qilu Normal University, Jinan 250200, China

**Keywords:** poplar wood, formic acid–phloroglucinol fractionation, in situ conversion, heterocyclic compounds, hydrophobic fabrics

## Abstract

During the conventional biomass fractionation, the degradation and dissolution of lignin and hemicellulose result in a complex extract which remains very challenging for the thorough separation and purification of a wide variety of fractionated products, limiting their further utilization. Herein, we proposed a facile and efficient strategy for fractionating biomass and simultaneously in situ converting of both lignin and hemicellulose into single products using a formic acid–phloroglucinol system. The introduced phloroglucinol could react with lignin fragments and hemicellulose-derived products, and the generated intermediate product from hemicellulose can be further condensed with lignin fragments, finally forming single lignin-based functional biopolymers containing heterocyclic structures. Only small amounts of hemicellulosic derivatives, such as oligosaccharides, monosaccharides, furfural, and 5-HMF, were detected in the extracted solution, indicating a highly directional and effective in situ conversion process of hemicellulose. The constructed specific structures on fabric surfaces by using the chelation between lignin-based functional biopolymers and metal ions achieved the preparation of functional fabrics with stable hydrophobicity. The dynamic contact angle of water droplets on the surface of prepared fabric only decreased from 122° to 116.8° over 30 min. This work strategy provides an ideal route to maximize the utilization of both lignin and hemicellulose without involving complex separation and purification procedures. This strategy is the first demonstration of using the targeted fractionation system to achieve the simultaneous conversion of hemicellulose and lignin into single functional biopolymers directly from lignocellulosic biomass.

## 1. Introduction

With the increasing depletion of fossil energy and the continuous aggravation of environmental problems, the demand for sustainable and renewable resources has become more and more urgent [1,2]. Against this background, lignocellulosic biomass, as a widely existing, renewable, and environmentally friendly resource, has increasingly captured the attention of the scientific community and industrial world [3]. Lignocellulosic biomass mainly consists of lignin, carbohydrates (mainly cellulose and hemicellulose), as well as a small amounts of extracts and ash. Lignin, a complex three-dimensional network aromatic polymer, possesses abundant functional groups and various types of linkages, endowing lignin unique chemical and physical properties, such as hydrophobicity, ultraviolet (UV) resistance, and environmental stability [4,5,6,7,8,9,10]. Cellulose presents a linear high-molecular-chain structure, which is composed of glucose units connected by β-1,4 glycosidic bonds, possessing good crystallinity and mechanical strength [8,11,12,13,14]. Hemicellulose is a kind of complex heteropolysaccharide, usually containing a variety of monosaccharide units, such as xylose, arabinose, mannose, etc. [15,16]. Hemicellulose is relatively unstable due to its low degree of polymerization, which is easily hydrolyzed and converted into various derivative products [12,13,17]. Based on the utilization promising of main components, lignocellulosic biomass has been regarded as a substitute for fossil resources to produce biofuels, biobased materials, and chemicals.

Traditional pathways of utilizing biomass primarily focus on the separation and simple processing of a single component. In current manufacturing processes of chemical pulp and dissolving pulp, cellulose was extracted from wood for paper production, while large amounts of lignin and hemicellulose were burned to recover heat energy, emitting massive amount of harmful greenhouse gases [18,19,20]. The novel fractionation processes, including Organoslv fractionation, ionic liquid pretreatment, and deep eutectic solvents (DES) fractionation, have also developed to promote the valorization of the whole components of biomass [21,22,23]. Some fractionation technologies often rely on extreme conditions, such as high temperature, high pressure, and strong acids and alkalis, to strengthen the removal degree of lignin and hemicellulose. Under such harsh conditions, lignin and hemicellulose tend to suffer excessive degradation and repolymerization reactions, limiting the further valorization of the produced products [24,25,26,27,28,29,30]. During the fractionation process, stabilization strategies, using chemical functionalization of reactive intermediates, have been designed to suppress the undesirable condensation of lignin and adjust the disordered depolymerization of polysaccharides [31,32,33]. These strategies open the window for directly upgrading the produced biopolymers, such as lignin or hemicellulosic derivatives, to well-defined platform molecules. However, the degradation and dissolution of lignin and hemicellulose after lignocellulosic biomass fractionation resulted in a mixture consisted of complex products, including lignin fragments with different molecular weight, various types of sugars and furans, which posed fundamental challenges to industrial separation and purification processes.

Currently, studies have shown that lignin degradation products (phenolic compounds) and carbohydrate derivatives (sugars and furans) can be combined through chemical or biological pathways to manufacture bio-based materials [34,35]. Under specific degradation conditions, hemicellulose degradation can yield a series of active small-molecule products, such as furfural and 5-hydroxymethylfurfural (5-HMF). Moreover, the functional groups endow lignin structure with abundant active sites that could cause the reaction with carbohydrate-derived compounds [36]. For example, the aldehyde group in the furfural molecule can undergo a nucleophilic addition reaction with the phenolic hydroxyl group on the phenylpropane unit of lignin to form a product connected by ether bonds with a stable structure [37]. Furfural molecules can also condense with lignin to construct functional biopolymers containing heterocyclic structures that enable materials with both rigidity and stability [38,39]. The composite materials prepared by combining carbohydrate degradation products with lignin remain the fundamental properties of both, contributing to break through the performance bottlenecks of traditional materials. However, these combination processes for preparing composites are generally achieved based on the perfect finished product obtained after various processing pathways of lignocellulosic biomass, such as Kraft lignin and furfural. Therefore, the in situ combination of lignin and hemicellulosic derivatives during biomass fractionation not only greatly shorten the whole manufacturing process but is also beneficial to address the fundamental challenges to separate and purify the fractionated products from the complex extracts.

The phenolic hydroxyl groups in lignin structure could form stable complexes with metal ions (Cu^2+^, Fe^3+^, Ca^2+^) through ion exchange and surface interactions across pH 3–9 [40,41]. This enables efficient metal chelation (50–200 mg/g) for improving textile dyeing and effective heavy metal removal (>95% Pb^2+^) [42,43,44]. Importantly, the efficient metal chelation endows lignin with excellent hydrophobic ability. Lignin phenolic -OH groups crosslinking with Fe^3+^/Cu^2+^ could create stable 3D networks that immobilize hydrophobic aromatic structures [45]. These innovative approaches contribute to address traditional biomaterial challenges, facilitating the development of sustainable multifunctional textiles [46]. Herein, we proposed a new strategy for realizing the in situ conversion of lignin and hemicellulose into single functional biopolymers during the fractionation process of lignocellulosic biomass. Specifically, we innovatively introduced a fractionation system composed of formic acid and phloroglucinol to fractionate the main constituent of poplar wood and simultaneously achieve the in situ combination of the extracted lignin and hemicellulosic derivatives. Under the acidic environment, hemicellulose-derived products reacted with phloroglucinol and the generated intermediate product could further react with the lignin fragments, finally forming the functional biopolymers containing heterocyclic structures. After detailed component measurement, only small amounts of hemicellulosic derivatives, such as oligosaccharides, monosaccharides, furfural, and 5-HMF, were detected in the extracted solution, indicating a highly directional and effective reaction process for the degradation and transformation of carbohydrates. Based on the in-depth characteristic investigate of these heterocyclic compounds, we boldly attempted to apply the extract to the synthesis field of hydrophobic fabrics by metal ion chelation. The fabrics prepared showed excellent hydrophobic properties, which opens a new path for the development of functional textiles. To best of our knowledge, the proposed strategy is the first demonstration of using the targeted fractionation system to achieve the simultaneous conversion of hemicellulose and lignin into single products directly from lignocellulosic biomass. This cutting-edge approach is expected to address fundamental challenges in traditional biomass utilization and achieve efficient transformation and full resource utilization.

## 2. Materials and Methods

### 2.1. Materials

Poplar chips were crushed into powder, and 40–60 mesh was selected. The detailed analysis of the main components was conducted according to NREL standards [47]. Phloroglucinol (≥98%), formic acid (85%), guaiacylglycerol-β-guaiacyl ether (GG), D-xylose (>90%) and 5-HMF (≥99.5%) were purchased from Aladdin Bio-Chem Technology (Shanghai, China). Formic acid (88%), furfural (99%), ethyl acetate (99%), acetonitrile (≥99.9%) chloroform (99%), and iodine (99.8%) were purchased from Shanghai Macklin Biochemical Technology Co., Ltd. (Shanghai, China). N-hexane (≥99.5%) were purchased from Tianjin Fuyu Fine Chemical Co., Ltd. (Tianjin, China). All chemicals were analytical grade or above and used without further purification.

### 2.2. Formic Acid–Phloroglucinol Treatment

**Figure 1:**
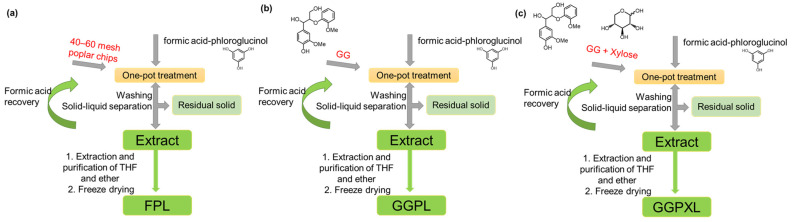
The phloroglucinol–formic acid separation process of poplar (**a**), GG (**b**), and GG with Xylose (**c**).

15 g of poplar powder and 150 mL of co-solvent (a certain ratio of formic acid and phloroglucinol, abbreviated as F:P here in after) were intensively mixed in the Teflon—lining of hydrothermal reactor) were intensive mixed in the Teflon lining of the hydrothermal reactor. The reactor was heated to 100 °C, 120 °C, and 140 °C using oil-bath heating. After reaching the reaction time, the reactor was cooled to room temperature, and then the reacted mixture was filtered. The obtained residue solid was washed with 200 mL of formic acid and then fully washed with deionized water (DI water). The filtrate and formic acid washing liquid were mixed, and the resulting solution was regarded as the extracted solution. The solvents in the extracts were recovered using vacuum rotary evaporation and obtained concentrated solution. Tetrahydrofuran was added to the concentrated solution. After centrifugation, the supernatant was dripped into ether and the precipitate was obtained by centrifugation. After further drying, the purified lignin products were obtained and the lignin samples from the treatment at 100 °C, 120 °C, and 140 °C were marked as FPL100, FPL120, and FPL140, respectively (Figure 1a). As the control, the formic acid treatments without phloroglucinol addition were carried out, and the lignin samples obtained at 100 °C, 120 °C, and 140 °C were marked as FL100, FL120, and FL140, respectively.

In order to reveal the reaction mechanism of hemicellulose and lignin in the formic acid–phloroglucinol treatment system, xylose and GG were used as the models of hemicellulose and lignin, respectively, to carry out the above process. Using GG as raw materials, the extraction process was treated with formic acid and phloroglucinol, and the yielded extraction liquids were designated as GGPL (Figure 1b). Using GG and xylose as the mixed raw materials, the extraction process was treated with formic acid and phloroglucinol, and the yielded extraction liquids were designated as GGXPL (Figure 1c).

### 2.3. Characterization of Extracted Products

The content of hemicellulosic sugars in the extracts was analyzed by high-performance liquid chromatography (HPLC) that used a Thermo Scientific ICS-5000+ HPLC system equipped with a Dionex CarboPac Bio-LC column (3 × 150 mm) at 80 °C. The mobile phase consisted of water and 50 mmol/L NaOH, with a flow rate of 0.4 mL/min over 52 min. A Thermo Scientific Ultimate 3000 RSLC system, equipped with a UV-Vis detector and a Polar Advantage II C18 column (30 °C), was used for the detection of furfural and 5-HMF. The mobile phase consisted of 10% methanol in water, with isocratic elution at a flow rate of 0.8 mL/min for 20 min. Gas Chromatography-Mass Spectrometry (GC-MS) were carried out for identifying the products by using an Agilent 9000C GC with an HP5-MS capillary column and a 5977A mass spectrometer. The temperature program was as follows: 50 °C for 0.5 min, ramp to 225 °C at 10 °C/min, and hold for 15 min (total run time: 33 min). The ^13^C-^1^H short range correlation experiment was performed using the Bruker standard pulse program hsqcedetgpsisp2.2 (phase sensitive, gradient-edited, sensitivity enhanced 2D-HSQC using adiabatic pulses for inversion and refocusing) using non-uniform sampling of 50%. Spectra were acquired using 40 scans and an interscan delay of 1s for a total acquisition time of 3 h with a 12 ppm sweep width in ^1^H using 1024 data points for an acquisition time of 85 ms; and in ^13^C, 215 ppm sweep width using 512 increments for acquisition time of 9.74 ms. Data processing used squared cosine-bell in ^1^H and ^13^C resulting in a 1024 × 1024 data matrix. Topspin 3.7p17 was used for interactive integration of 2D cross-peaks [48,49].

Thin-layer chromatography (TLC) was performed on Millipore precoated silica gel plates (0.20 ± 0.03 mm, particle size 8 ± 2 μm) using a Hexane/Chloroform/EtOAc (2:1:2) solvent system to monitor the reaction. After completion, the silica gel was separated by centrifugation, and the solid residue was washed with acetonitrile (2 × 2 mL). The combined organic solvent was evaporated under vacuum to obtain the products that were further characterized.

### 2.4. Preparation of FPL\Fe^3+^\Fabric

The cotton fabric was cut into a circle with a diameter of 7 cm and washed with deionized water, anhydrous ethanol, and deionized water for 5 min. The 25 mL of 3 g/L FPL120 solution was mixed with 25 mL of 3 g/L FeSO_4_·7H_2_O solution, FeCl_3_ solution, and CuSO_4_ solution, respectively, magnetic stirring 450 r/min for 5 min at room temperature. The cotton fabric was immersed in a mixed solution and remained at 40 °C for 24 h. The cotton fabric was taken out, the lignin/metal particles were deposited on the cotton fabric, and the surface was turned every 4 h to ensure uniform deposition. Finally, FPL120\Fe^2+^\fabric, FPL120\Fe^3+^\fabric, FPL120\Cu^2+^\fabric were obtained by washing with deionized waterand drying at 45 °C for 6 h. A total of 25 mL of FPL100 solution and FPL140 solution with a concentration of 3 g/L were mixed with 25 mL of 3 g/L FeCl_3_ solution at room temperature, and the above process was carried out. FPL100\Fe^3+^\fabric and FPL140\Fe^3+^\fabric was obtained, respectively.

### 2.5. Characterization of Hydrophobic Properties of Modified Fabric

The water contact angle (WCA) of the fabric before and after modification was evaluated using the OCA50 (DATAPHYSICS.OCA50, Filderstadt, Germany) contact angle tester. The volume of water is 5 μL, and the contact angle is measured five times to take the average value. X-ray photoelectron spectroscopy (XPS, Thermo Scientific K-Alpha, Waltham, MA, USA) was used to test the surface chemical composition of the fabric before and after modification. The spectrometer was equipped with a monochromatic X-ray source (Al Kα X-ray source; hν = 1486.6 eV, 6 mA, 12 kV)

**Figure 2:**
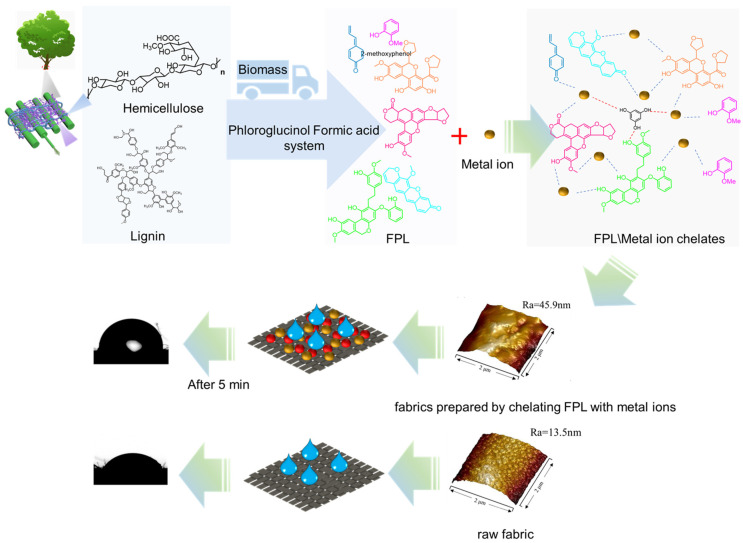
Direct in situ conversion of both lignin and hemicellulose into functional biopolymers for preparing hydrophobic fabrics.

Figure 2 shows the direct in situ conversion of both lignin and hemicellulose into functional biopolymers for preparing hydrophobic fabrics.

## 3. Results and Discussion

### 3.1. The Analysis of Formic Acid–Phloroglucinol Treatment

**Figure 3:**
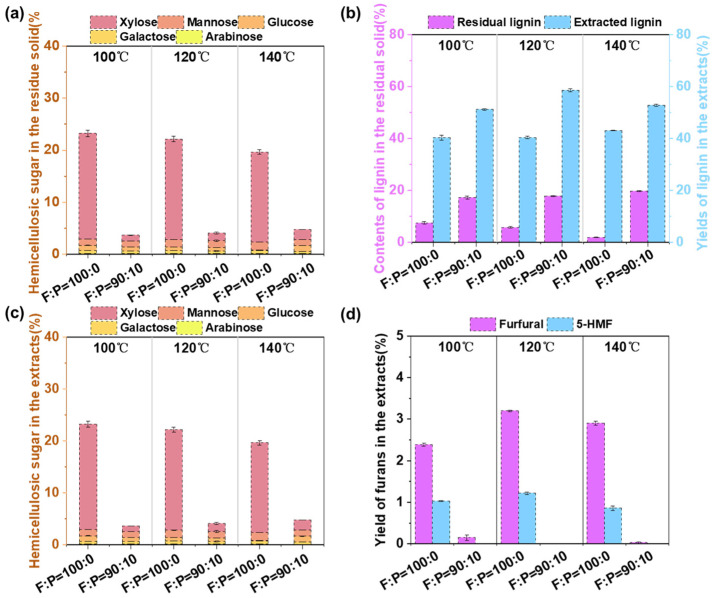
Composition analysis after the fractionation process without and with the addition of phloroglucinol. (**a**) the content of hemicellulosic sugars in the residue solid. (**b**) the lignin content remained in the residue solid and extracted solution. (**c**) the content of hemicellulosic sugars in the extracts. (**d**) the content of furfural and 5-HMF in the extracts.

The fractionation process of biomass components plays a critical role on the subsequent purification and utilization. Figure 3 illustrates the effect of formic acid treatment with or without phloroglucinol addition on the component separation of lignocellulosic biomass. As shown in Figure 3a, the addition of phloroglucinol had a positive impact on the removal rate of hemicellulose in the residual solid under various reaction temperatures. Under a reaction temperature of 100 °C, the residual solid obtained through formic acid treatment contained 23.06% hemicellulosic sugars, while that of residue solid obtained from the formic acid–phloroglucinol system contained 19.63%. With the increasing of temperature, the yield of hemicellulosic sugar in the residual solid exhibited a slight decreasing trend. Specifically, the sugar yields in the residual solid treated with formic acid decreased from 23.06% to 16.65%, while that in the solids treated with the formic acid–phloroglucinol system decreased from 19.63% to 14.38%. These results suggest that the addition of phloroglucinol in the formic acid treatment exhibited a positive influence on the removal of hemicellulose. Figure 3b shows the content of lignin in the residual solid and the yield of extracted lignin products after formic acid treatment with or without phloroglucinol. During the formic acid treatment without phloroglucinol, the results indicate that the remained lignin in residual solid decreased as the temperature increases, accordingly, the yield of lignin products in the extracts increased from 39.70% to 43.05%. This trend is due to higher reaction temperature could promote the efficient removal of hemicellulose and lignin from lignocellulosic biomass. However, with the addition of phloroglucinol, the contents of lignin in the residual solid and extracted lignin products showed a surprisingly high yield, which of sum far exceeded the lignin content in the initial raw material. Additionally, both the residual substrates and obtained lignin products from formic acid–phloroglucinol treatment exhibited varying degrees of red coloration (Figure S1a). These results suggested that phloroglucinol may have reacted with the lignocellulosic components in the residual solids or dissolved in the extracts.

To further verify the effect of the added phloroglucinol on the biomass separation, the degraded hemicellulosic compositions in the extracts were measured. Figure 3c shows a significant effect of the phloroglucinol addition on the yield of hemicellulosic sugars in the extracts. With increasing reaction temperature, the sugar yields in the extracts obtained from formic acid treatment decreased from 23.22% to 16.65%, which may be due to the dissolved sugars experiencing a further dehydration reaction and forming furans in the acidic environment [50]. In contrast, with the addition of phloroglucinol, the extraction treatment system led to a significant reduction of sugar yields. Under the reaction temperature of 100, 120 and 140 °C, the formic acid–phloroglucinol treatment yielded 3.68%, 4.12% and 4.78% of hemicellulosic sugars, respectively, which were reduced by 84.15%, 81.41% and 75.76%, respectively, compared with formic acid treatment. Figure 3d presents the yields of two major degradation products (furfural and 5-HMF) in the extracts. For formic acid treatment, the yields of furfural and 5-HMF were lowest at 140 °C (2.41% and 0.85%, respectively) and highest at 120 °C (3.21% and 1.22%, respectively). In contrast, when phloroglucinol was added, the maximum yields of furfural in the extract were only 0.15%, and 5-HMF was not detected. We can infer that the hemicellulosic degradation products extracted from lignocellulosic biomass not only underwent partial degradation in the acidic environment but may also have reacted with phloroglucinol to form complex products. Furthermore, the bright red color observed in the extracts from formic acid–phloroglucinol treatment (Figure S1b) supports the hypothesis that hemicellulosic derivatives may undergo complex reactions with phloroglucinol.

### 3.2. The Conversion Mechanism of the Formic Acid–Phloroglucinol System

**Figure 4:**
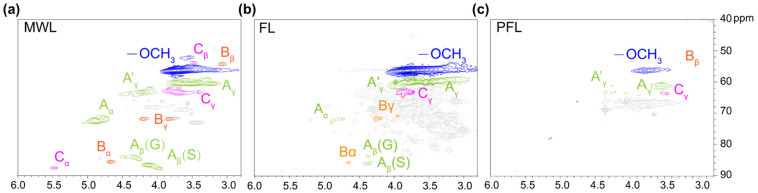
Aliphatic region of 2D HSQC NMR for MWL (**a**), FL (**b**), FPL (**c**).

**Figure 5:**
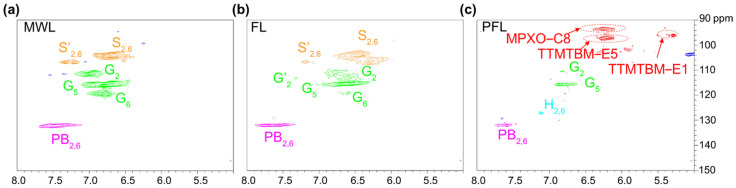
Aromatic region of 2D HSQC NMR for MWL (**a**), FL (**b**), FPL (**c**); MPXO: 12-methoxy-2H,9H-pyrano [3,2-b] xanthen-9-one, TTMTBM: (tetrahydrofuran-2-yl)(1,3,9-trihydroxy-8-methoxy-6-(tetrahydrofuran-3-yl)-6H-benzo[c]chromen-4-yl)methan one.

**Figure 6:**
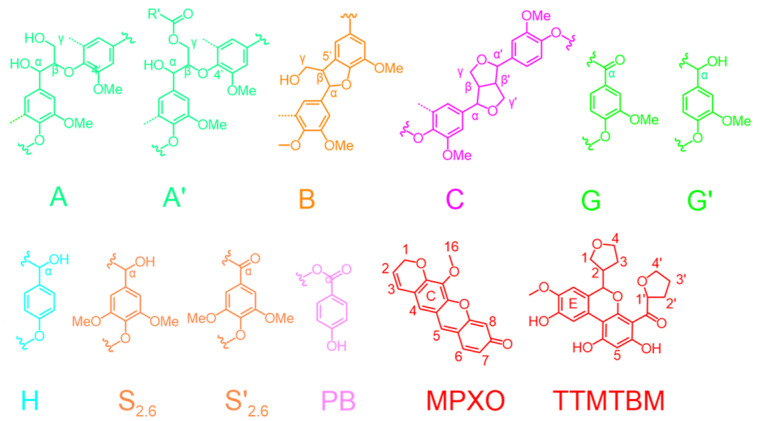
Main structures identified in the lignins: A, β-O-4 aryl ether linkages with a free-OH at the γ-carbon; A′,Cα-oxidized β-O-4 aryl ether linkages; B, resinol substructures formed by β-β, α-O-γ,and γ-O-α linkages; C, phenylcoumarane substructures formed by β-5 and α-O-4 linkages; G, guaiacyl units; G′, oxidized guaiacyl units with a Cα-ketone or a Cα carboxyl group; H, p-hydroxyphenyl units, S_2.6_, syringyl units; S′_2.6_, oxidized syringyl units with a Cα ketone or a Cα carboxyl group; and PB, p-hydroxybenzoate substructures.

Both the content and chemical composition of extracted products from poplar exhibited significant difference after the formic acid treatment process with or without the addition of phloroglucinol. To reveal the possible reaction mechanism between phloroglucinol and extracted fractions, we first studied the structural features of the obtained lignin products. Figure 4 shows the aliphatic region of 2D HSQC NMR for milled wood lignin (MWL), lignin obtained by formic acid treatment at 120 °C (FL), and lignin obtained by the formic acid–phloroglucinol treatment at 120 °C (FPL), displaying two main regions corresponding to aliphatic (approximately δC/δH 40–90/2.5–6.0 ppm) and aromatic (approximately δC/δH 100–150/5.0–8.0 ppm) ^13^C-^1^H correlations [51]. By comparing Figure 4, it can be clearly observed that there are significant traces of carbohydrates in the aliphatic region of FL. However, in the same aliphatic region of FPL, the signal intensity is obviously decreased. This result is consistent with the analysis conclusions of Figure 3b,d, indicating that the addition of phloroglucinol promoted the hydrolysis of carbohydrates and effectively facilitated the cleavage of glycosidic bonds between lignin and hemicellulose. Figure 5 shows the aliphatic region of 2D HSQC NMR for MWL, FL, and FPL. Further comparing Figure 5, it can be found that there is no significant structural change in the aromatic region of FL and MWL, which means that formic acid has a relatively small impact on the benzene ring structure of lignin. Notably, in the aromatic region of FPL, the S- and G-type structural units are significantly damaged, and an unidentified signal appears in the range of δC/δH 90/6.0 ppm—105/6.5 ppm. Based on this phenomenon, we inferred that the intervention of phloroglucinol may destroy the original S- and G-type structural units in lignin and simultaneously promote the formation of new structures, thus resulting in the generation of the new signal in the region of δC/δH 90/6.0 ppm-105/6.5 ppm. Table 1 presents the phenolic hydroxyl content of FPL and MWL. The results indicate that the total phenolic hydroxyl content of FPL is higher than that of MWL at 100–140 °C. Importantly, the significantly enhanced phenolic hydroxyl groups in H-unit of lignin structure was the most notable feature compared with MWL. It can be inferred that the introduced phloroglucinol might have reacted with the lignin structure; thus, the grafted phloroglucinol increased the content of hydroxyl groups. Figure 6 shows the main structures identified in the lignins.

**Figure 7:**
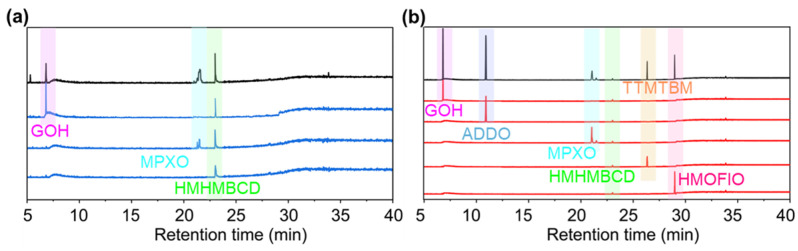
GC-MS chromatogram of GGPL (**a**) and GGPXL (**b**).

**Figure 8:**
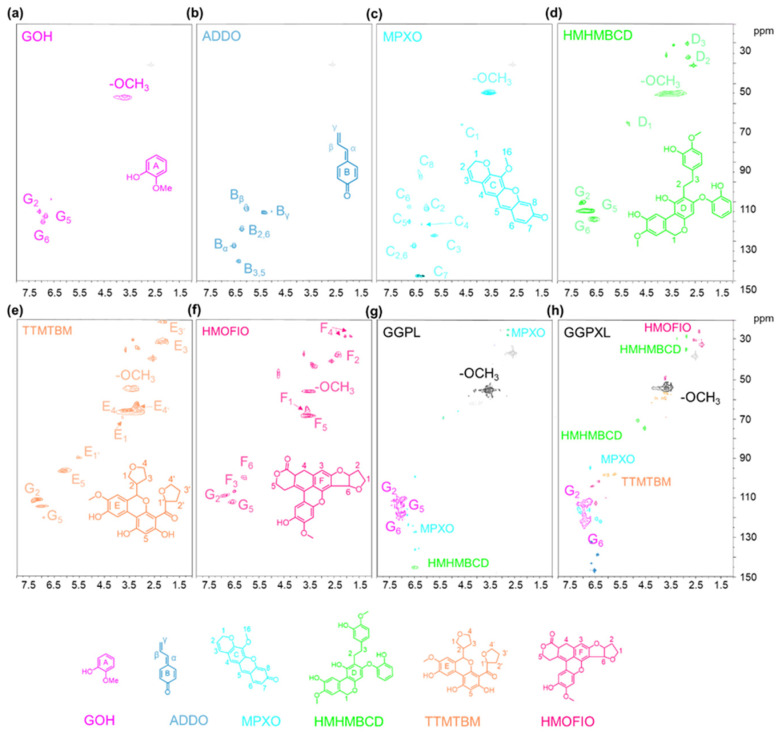
The structural features of the purified products (**a**) GOH; (**b**) ADDO; (**c**) MPXO; (**d**) HMHMBCD; (**e**) TTMTBM; (**f**) HMOFIO separated from GGPL and GGPXL characterized by 2D-HSQC NMR, and (**g**) GGPL; (**h**) GGPXL.

In order to further understand the reaction mechanism, Glycerol-β-guaiacyl ether (GG), a phenolic lignin dimer with methoxy substituents on each benzene ring, was used as a model to represent the complex lignin structure. Xylose was used as a model of hemicellulose to reveal the reaction mechanism during the formic acid–phloroglucinol treatment. As the raw materials, the GG was introduced in the formic acid and phloroglucinol treatment system, as shown in Figure S2a, and the yielded extraction liquid was designated as the GGPL solution. To investigate the reactions between phloroglucinol and the degradation products of lignin and hemicellulose, GG and xylose were introduced into the reaction system together, as shown in Figure S2b, with the yielded extraction liquids designated as the GGXPL solution. After further isolation and purification of the obtained two solutions, the produced products were speculated via GC-MS (Figure 7 and Figure S2) and 2D-HSQC NMR (Figure 8) analysis [33,52]. As shown in Figure 7a,b, three products were speculated in both GGPL and GGXPL solutions, including Guaiacol (GOH, Figure 8a), MPXO (Figure 8c), and 3-hydroxy-2-methoxy-5,6,8a,9,11a,12,13,14a-octahydro-8H-furo[2′,3′:4,5]furo[2,3-c]isochromeno[5,6,7-mn]xanthen-8-one (HMHMBCD, Figure 8d), which had been successfully separated. Additional novel compounds were speculated in the GGXPL GC-MS chromatogram. Based on our analysis, we propose these compounds to be 4-allylidenecyclohexa-2,5-dien-1-one (ADDO, Figure 8b), TTMTBM (Figure 8e), and HMOFIO (Figure 8f). These purified products, separated from GGPL and GGXPL solution, were further characterized by detecting their structural features using 2D-HSQC NMR. As shown in Figure 8a–f, the related signals showed in the spectra of 2D-HSQC NMR could be attributed to the typical linkages of the six products speculated by GC-MS, further confirming the speculate compounds of the output products obtained from the formic acid–phloroglucinol treatment. These results indicated that the part of degradation products from lignin and hemicelluloses could react with phloroglucinol during the fractionation process. According to the above speculate products from the treatment of models, their structural features could be further confirmed in the 2D-HSQC NMR spectra of the produced lignin products from the treatment of poplar wood (Figure 5c). Based on Figure 8c,e, we speculate that the signal region of δC/δH 92/6.47 ppm, δC/δH 98.2/6.38 ppm, and δC/δH 95.3/5.34 ppm could be attributed as **MPXO**−**C8**, **TTMTBM**−**E1** and **TTMTBM**−**E5**, respectively. Based on the speculated products from the model reaction and the structural features of the obtained lignin products from poplar wood, we speculate that the introduced phloroglucinol could react with lignin fragments and hemicellulosic derivatives, especially furfural, during the formic acid–phloroglucinol fractionation process. Importantly, the TTMTBM structures presented in lignin products indicated that the compound generated from the reaction between phloroglucinol and furfural occurred from the condensation with lignin, forming the lignin-based functional biopolymers containing heterocyclic structures. Therefore, the designed formic acid–phloroglucinol fractionation system not only extracted lignin and hemicellulose from biomass with high efficiency but also achieved the in situ conversion of dissolved lignin fragments and hemicellulosic derivatives into single compounds with heterocyclic structures, which greatly contributed to the subsequent separation and purification of products from extracts.

### 3.3. Hydrophobic Fabrics Were Prepared by Combining Hemicellulose and Lignin with Metal Ions

**Figure 9:**
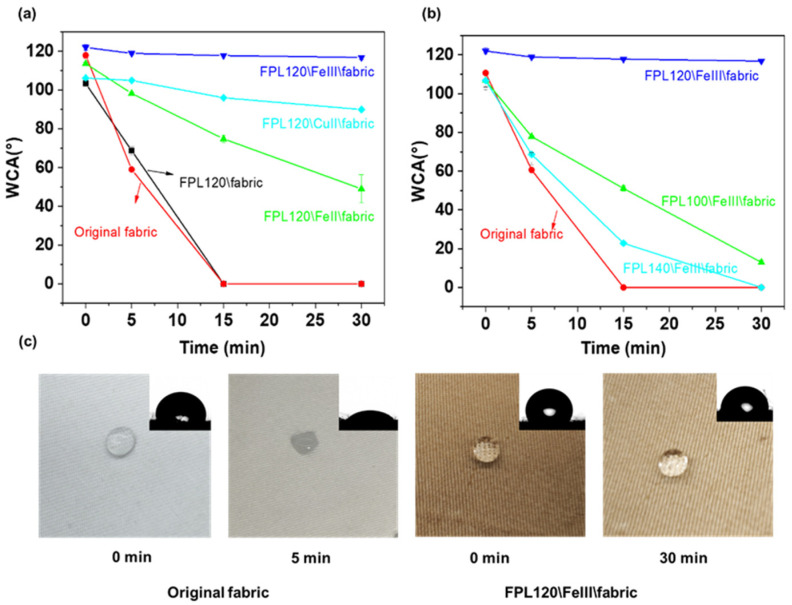
(**a**) The water contact angles (WCA) of fabrics prepared by chelating FPL120 with three different metal ions over a time range of 0–30 min. (**b**) The WCA of fabrics prepared by chelating Fe^3+^ with FPL100, FPL120, and FPL140 over a time range of 0–30 min. (**c**) The images of water droplets on the raw fabric and surface modified fabric.

The regulation of material surface properties through the interaction of specific chemical substances has become a key research focus in the field of surface modification. Lignin, due to their variable structural features, can chelate with metal ions to form orderly cyclic complexes. This chelation process can lead to the formation of uniformly distributed complexes on the material surface, which contributes to a relatively uniform surface roughness, thereby achieving the adjustment of the material surface property [53,54]. The surface roughness has a significant influence on the hydrophobicity of materials, and the combination of appropriate roughness with low-surface-energy substances can impart excellent hydrophobic properties to the material [55]. Figure 9a shows the water contact angles (WCA) of fabrics prepared by chelating FPL120 with three different metal ions over a time range of 0–30 min. Among the three metal ions, the fabric prepared by chelating FPL120 with Fe^3+^ exhibited the best hydrophobicity. Over 30 min, the WCA of fabric prepared by the chelation of FPL120 with Fe^3+^ decreased from 122° to 116.8° (Figure 9a,b), showing the relatively stable hydrophobicity. In contrast, both the raw fabric and the surface-modified fabric using FPL120 without chelation showed poor hydrophobicity, and the water droplets have completely penetrated the fabrics with 15 min (Figure 9a,c). Compared with lignin-based oxygen-containing heterocyclic compounds obtained from various reaction temperatures, the surface modified fabric using FPL120 possessed stable hydrophobicity (Figure 9b). It can be observed that the hydrophobic performance of the fabrics is negatively correlated with the phenolic hydroxyl content of FPL (as shown in Table 1), indicating that lignin-based oxygen-containing heterocyclic compounds with less content of phenolic hydroxyl groups contributed to the enhancing of material hydrophobicity. Therefore, the constructed specific structures on fabric surfaces by using the chelation between lignin-based oxygen-containing heterocyclic compounds and metal ions not only achieved the preparation of functional fabrics with stable hydrophobicity but also strengthened the promising of the products from formic acid–phloroglucinol fractionation process.

**Figure 10:**
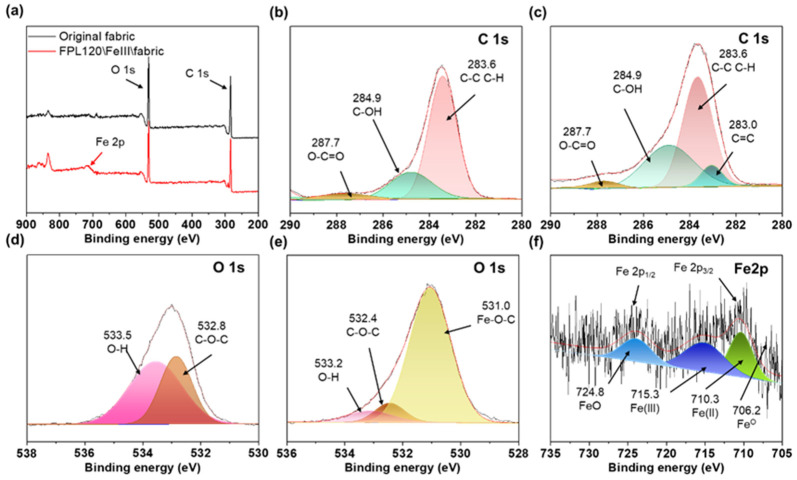
(**a**) The XPS spectrum of the original fabric and FPL120\Fe^3+^\fabric. The high-resolution C 1s (**b**) and O 1s (**d**) of the original fabric, and the high-resolution C1s (**c**), O1s (**e**) and Fe2p (**f**) spectra of the FPL120\Fe^3+^\fabric.

As can be seen in the XPS spectrum (Figure 10a), the peak of Fe2p observed at 711 eV on the FPL120\Fe^3+^\fabric indicates the presence of iron on the fabric. The C1s high-resolution spectra of the original fabric and FPL120\Fe^3+^\fabric presented the peaks at 283.6, 284.9, and 287.7 eV, corresponding to the C atoms in C-C or C-H, C-OH, and O-C=O bonds, respectively (Figure 10b,c) [56,57]. Quantitative analysis of surface elemental content was performed by calculating the peak areas, and the results are summarized in Table 2. Due to the addition of FPL and Fe^3+^, the content of C-H and O-C=O groups on the FPL120\Fe^3+^\fabric surface decreased from 69.78% and 39.45% to 53.83% and 8.54%, respectively, while the content of C=C and C-OH increased from 0% and 23.08% to 7.43% and 34.61%, respectively. These results confirmed that FPL120 formed a chelate with Fe^3+^ and adhered to the cotton fabric surface through hydrogen bonding. Figure 10d,e displays the O1s high-resolution spectra of the original fabric and FPL120\Fe^3+^\fabric, respectively. We could observe that the peaks at 532.4 and 533.5 eV corresponded to the O atoms in C-O-C and O-H bonds [58], and a new sub-peak observed at 531.0 eV in the FPL120\Fe^3+^\fabric spectrum (Figure 10e) represented the formation of Fe-O-C bonds, confirming the chelation between FPL and Fe^3+^. Additionally, the appearance of Fe2p peaks in Figure 10f further demonstrated the loading of Fe on the fabric surface.

### 3.4. Feasibility Analysis of FPL120\/Fe^3+^ Fabric

**Figure 11:**
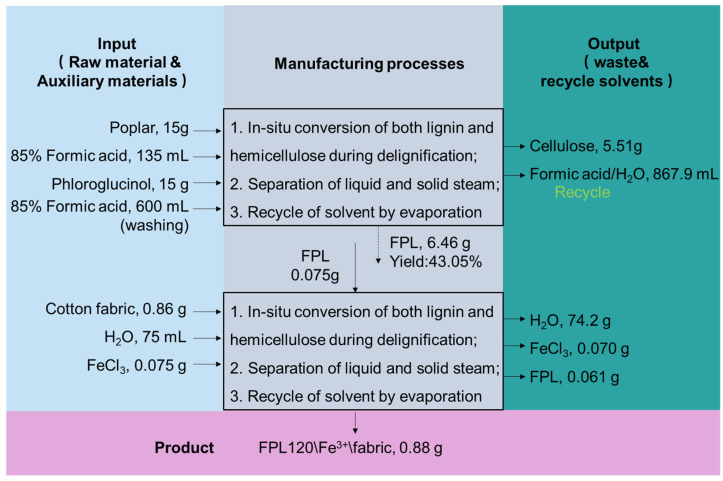
Mass flow of the conversion process for biomass to hydrophobic fabrics.

As shown in Figure 11, the feasibility analysis was conducted on the FPL120\Fe^3+^ functional fabric using green chemistry indicators. The systematic comparison of three representative materials (FAL\Cu, FAL\Fe, and CuO-NPs fabrics) is shown in Table 3. Key metrics including *E*-factors (*E*simple and *E*complex), Mass Intensity (MI), and Reaction Mass Efficiency (RME) were analyzed to evaluate the environmental friendliness and sustainability performance of these materials. Comparative analysis of FPL120\Fe^3+^ fabric against FAL\Cu, FAL\Fe, and CuO-NPs showed that FPL120\Fe^3+^ achieved an *E*_simple_ of 0.84 (between FAL\Cu (0.14) and CuO-NPs (1.10)), indicating moderate reactant efficiency. Its *E*_complex_ (96.54) is higher than that of CuO-NPs (115.9) and comparable to FAL\Fe (98.62), demonstrating superior solvent economy versus nanoparticles. While MI (98.52) was higher than that of the FAL-series, its remarkable 68.4% RME significantly exceeded FAL\Cu (40%) and FAL\Fe (56%). These results demonstrated that FPL120\Fe^3+^ could be regarded as an environmentally favorable bio-based material due to its competitive process efficiency and outstanding biomass utilization for functional textile production.

## 4. Conclusions

This work demonstrated a new strategy for realizing in situ conversion of lignin and hemicellulose into single functional biopolymers by using the formic acid–phloroglucinol fractionation process of lignocellulosic biomass. The results showed that both hemicellulose and lignin were extracted from poplar wood with high efficiency during the fractionation process. Remarkably, the introduced phloroglucinol could react with the extracted lignin and hemicellulosic derivatives. Moreover, the lignin fragments also occurred condensation with intermediate product generated from the hemicellulosic derivatives and derivatives. These reaction routes finally formed single lignin-based functional biopolymers containing heterocyclic structures. The detailed component measurement exhibited that only small amounts of hemicellulosic derivatives were detected in the extracted solution, indicating a highly directional and effective transformation process of hemicellulose. The produced lignin-based heterocyclic compounds were chelated with metal ion and further used to the synthesis of hydrophobic fabrics. The proposed strategy opens a window for enabling the simultaneous valorization of lignin and hemicellulose in a one-pot fractionation process without any further separation and purification, which is a huge advantage to realize industrial-scale production.

## Figures and Tables

**Table 1:** Phenolic hydroxyl contents of lignin samples obtained by quantified ^3l^P NMR.

	Hydroxyl Group (mmol/g)					
Sample	Aliphatic-OH	S-OH	G-OH	H-OH	Condensed-OH	Total Phenolic
MWL	4.49	0.21	0.70	0.61	0.01	1.53
FPL100	0.58	026	0.39	1.92	0.48	3.05
FPL120	0.56	0.3	0.37	1.62	0.40	2.70
FPL140	0.54	0.51	0.87	2.05	0.61	4.04

**Table 2:** The chemical structure content determined by XPS spectroscopy.

		Chemical Groups (%)					
Sample	C=C	C-C C-H	C-OH	O-C=O	C-O-C	Fe-O-C	O-H
Original fabric	-	69.75	23.08	7.17	39.45	-	60.45
FPL120\Fe^3+^\fabric	7.43	53.83	34.61	4.13	8.54	83.61	7.85

**Table 3:** Mass-based green metrics for evaluating sustainability for the whole process (the (*E*_simple_, **E**-factor calculated without any solvent other than the reactants; ***E***_complex_, *E*-factor calculated with solvents and reactants; MI, mass intensity; RME, reaction mass efficiency).

Process	*E*_simple_	*E*_complex_	MI	RME (%)	Ref
FPL120\Fe^3+^ fabric	0.84	96.54	98.52	68.4	This work
FAL\Cu fabric	0.14	89.69	85.69	98.93	[40]
FAL\Fe fabric	0.47	98.62	82.60	98.99	[55]
CuO-NPs fabric	1.10	115.9	88.95	101.71	[59]

## Data Availability

Data is contained within the article or Supplementary Materials. The data supporting this article have been included as part of the ESI.

## Supplementary Materials

The following supporting information can be downloaded at: https://www.mdpi.com/article/10.3390/polym17081029/s1, Figure S1. (a) Comparison of the color of residual substrates and lignin, (b) Comparison of the color of the extract. Figure S2. The mass spectrum of purified products separated from GGPL and GGPXL. Figure S3. The ¹H NMR and ^13^C NMR spectra for ADDO and MPXO. Figure S4. The products were obtained by separation and purification of VGPL (a) and VGPXL (b).

## References

[1] Kalogirou S.A. (2004). Solar thermal collectors and applications. Prog. Energy Combust. Sci..

[2] Zhou C.-H., Xia X., Lin C.-X., Tong D.-S., Beltramini J.J. (2011). Catalytic conversion of lignocellulosic biomass to fine chemicals and fuels. Chem. Soc. Rev..

[3] Abnisa F., Daud W.M.A.W. (2014). A review on co-pyrolysis of biomass: An optional technique to obtain a high-grade pyrolysis oil. Energy Convers. Manag..

[4] Feng Y., Li G.Y., Li X.Y., Zhu N., Xiao B., Li J., Wang Y.J. (2016). Enhancement of biomass conversion in catalytic fast pyrolysis by microwave-assisted formic acid pretreatment. Bioresour. Technol..

[5] Dou Z., Zhang Z., Wang M. (2022). Self-hydrogen transfer hydrogenolysis of native lignin over Pd-PdO/TiO2. Appl. Catal. B Environ..

[6] Wang Y., Liu Y., He J., Zhang Y. (2019). Redox-neutral photocatalytic strategy for selective C–C bond cleavage of lignin and lignin models via PCET process. Sci. Bull..

[7] Laurichesse S., Avérous L. (2014). Chemical modification of lignins: Towards biobased polymers. Prog. Polym. Sci..

[8] Zhang C., Wang F. (2020). Catalytic Lignin Depolymerization to Aromatic Chemicals. Acc. Chem. Res..

[9] Agarwal U.P., Ralph S.A., Padmakshan D., Liu S., Foster C.E. (2019). Estimation of Syringyl Units in Wood Lignins by FT-Raman Spectroscopy. J. Agric. Food Chem..

[10] Li Y., Shuai L., Kim H., Motagamwala A.H., Mobley J.K., Yue F., Tobimatsu Y., Havkin-Frenkel D., Chen F., Dixon R.A. (2018). An “ideal lignin” facilitates full biomass utilization. Sci. Adv..

[11] Zhang X., Lei H., Chen S., Wu J. (2016). Catalytic co-pyrolysis of lignocellulosic biomass with polymers: A critical review. Green Chem..

[12] Zhang B., Qiang G., Barta K., Sun Z. (2024). Bio–based polymers from lignin. Innov. Mater..

[13] Huang J., Fu S., Gan L. (2019). Chapter 2—Structure and Characteristics of Lignin. Lignin Chemistry and Applications.

[14] Wang Z., Deuss P.J. (2021). Catalytic Hydrogenolysis of Lignin: The Influence of Minor Units and Saccharides. ChemSusChem.

[15] Huang J., Yu L., Wang S., Qi L., Lu Z., Chen L., Xu D., Deng H., Chen C. (2023). An ultrathin nanocellulosic ion redistributor for long-life zinc anode. Innov. Mater..

[16] Wang Z., Xu C., Qi L., Chen C. (2024). Chemical modification of polysaccharides for sustainable bioplastics. Trends Chem..

[17] Xie J., Xu J., Zhang Z., Wang B., Zhu S., Li J., Chen K. (2023). New ternary deep eutectic solvents with cycle performance for efficient pretreated radiata pine forming to lignin containing cellulose nanofibrils. Chem. Eng. J..

[18] Sugiarto S., Leow Y., Tan C.L., Wang G., Kai D. (2022). How far is Lignin from being a biomedical material?. Bioact. Mater..

[19] Li C., Wu Y., Fu M., Zhao X., Zhai S., Yan Y., Zhang L., Zhang X. (2022). Preparation of Fe/N Double Doped Carbon Nanotubes from Lignin in Pennisetum as Oxygen Reduction Reaction Electrocatalysts for Zinc–Air Batteries. ACS Appl. Energy Mater..

[20] Reshmy R., Athiyaman Balakumaran P., Divakar K., Philip E., Madhavan A., Pugazhendhi A., Sirohi R., Binod P., Kumar Awasthi M., Sindhu R. (2022). Microbial valorization of lignin: Prospects and challenges. Bioresour. Technol..

[21] Zhang Y.C., Ni S.Z., Wu R.J., Fu Y.J., Qin M.H., Willför S., Xu C.L. (2022). Green fractionation approaches for isolation of biopolymers and the critical technical challenges. Ind. Crop. Prod..

[22] Liu Y.Z., Deak N., Wang Z.W., Yu H.P., Hameleers L., Jurak E., Deuss P.J., Barta K. (2021). Tunable and functional deep eutectic solvents for lignocellulose valorization. Nat. Commun..

[23] Amiri M.T., Dick G.R., Questell-Santiago Y.M., Luterbacher J.S. (2019). Fractionation of lignocellulosic biomass to produce uncondensed aldehyde-stabilized lignin. Nat. Protoc..

[24] Alriols M.G., Tejado A., Blanco M., Mondragon I., Labidi J. (2009). Agricultural palm oil tree residues as raw material for cellulose, lignin and hemicelluloses production by ethylene glycol pulping process. Chem. Eng. J..

[25] Nadif A., Hunkeler D., Käuper P. (2002). Sulfur-free lignins from alkaline pulping tested in mortar for use as mortar additives. Bioresour. Technol..

[26] Chen Y., Li Y., Zhang C., Qi H., Hubbe M.A. (2022). Holocellulosic fibers and nanofibrils using peracetic acid pulping and sulfamic acid esterification. Carbohydr. Polym..

[27] Olayo M.G., Alvarado E.J., González-Torres M., Gómez L.M., Cruz G.J. (2023). Quantifying amines in polymers by XPS. Polym. Bull..

[28] Li N., Li Y., Yoo C.G., Yang X., Lin X., Ralph J., Pan X. (2018). An uncondensed lignin depolymerized in the solid state and isolated from lignocellulosic biomass: A mechanistic study. Green Chem..

[29] Manzanares P. (2020). The role of biorefinering research in the development of a modern bioeconomy. Acta Innov..

[30] Sun S.-N., Li H.-Y., Cao X.-F., Xu F., Sun R.-C. (2015). Structural variation of eucalyptus lignin in a combination of hydrothermal and alkali treatments. Bioresour. Technol..

[31] Questell-Santiago Y.M., Galkin M.V., Barta K., Luterbacher J.S. (2020). Stabilization strategies in biomass depolymerization using chemical functionalization. Nat. Rev. Chem..

[32] Questell-Santiago Y.M., Zambrano-Varela R., Amiri M.T., Luterbacher J.S. (2018). Carbohydrate stabilization extends the kinetic limits of chemical polysaccharide depolymerization. Nat. Chem..

[33] Shuai L., Amiri M.T., Questell-Santiago Y.M., Heroguel F., Li Y.D., Kim H., Meilan R., Chapple C., Ralph J., Luterbacher J.S. (2016). Formaldehyde stabilization facilitates lignin monomer production during biomass depolymerization. Science.

[34] Li C., Zhao X., Wang A., Huber G.W., Zhang T. (2015). Catalytic Transformation of Lignin for the Production of Chemicals and Fuels. Chem. Rev..

[35] Wang H., Pu Y., Ragauskas A., Yang B. (2019). From lignin to valuable products–strategies, challenges, and prospects. Bioresour. Technol..

[36] Chang M., Wang X., Lin Q., Li R., Zhao L., Ren J., Zhang F.J. (2022). Formic acid–hydrogen peroxide treatment of furfural residue for production of nanocellulose, lignin, and nano-scale lignin. Green Chem..

[37] Zhan B., Zhang L., Deng Y., Yan L.J.G.C. (2023). A multifunctional lignin-based composite ultra-adhesive for wood processing. Green Chem..

[38] Qi H., Li Y., Zhou Z., Cao Y., Liu F., Guan W., Zhang L., Liu X., Li L., Su Y. (2023). Synthesis of piperidines and pyridine from furfural over a surface single-atom alloy Ru1CoNP catalyst. Nat. Commun..

[39] Liu J., Liu H., Chen L., An Y., Jin X., Li X., Liu Z., Wang G., Liu R.J. (2022). Study on the removal of lignin from pre-hydrolysis liquor by laccase-induced polymerization and the conversion of xylose to furfural. Green Chem..

[40] Liu X., Chen X., Bian H., Ni S., Li Z., Liu N., Qin M., Zhang F. (2023). Highly hydrophobic cotton fabric by in-situ co-deposition of lignin/metal particles for oil/water separation. Ind. Crops Prod..

[41] López-Maldonado E.A., Hernández-García H., Zamudio-Aguilar M.A.M., Oropeza-Guzmán M.T., Ochoa-Terán A., López-Martínez L.M., Martinez-Quiroz M., Valdez R., Olivas A. (2020). Chemical issues of coffee and Tule lignins as ecofriendly materials for the effective removal of hazardous metal ions contained in metal finishing wastewater. Chem. Eng. J..

[42] Chen F., Shahabadi S.I.S., Zhou D., Liu W., Kong J., Xu J., Lu X. (2019). Facile preparation of cross-linked lignin for efficient adsorption of dyes and heavy metal ions. React. Funct. Polym..

[43] Heo J.W., An L., Chen J., Kim M.S., Lee S.-D., Kim Y.S. (2022). Application of three types of aminated lignins for efficient removal of Cd(II) and Pb(II) ions in aqueous solution. BioResources.

[44] Zheng L., Seidi F., Wu H., Huang Y., Wu W., Xiao H. (2024). Low swelling Alginate/Lignin network gels with redox responsiveness for sustained release of agricultural fungicide and Pb2+ complexation. Eur. Polym. J..

[45] Chen H., Qu X., Liu N., Wang S., Chen X., Liu S. (2018). Study of the adsorption process of heavy metals cations on Kraft lignin. Chem. Eng. Res. Des..

[46] Gao Z., Duan L., Yang Y., Hu W., Gao G. (2018). Mussel-inspired tough hydrogels with self-repairing and tissue adhesion. Appl. Surf. Sci..

[47] Wu R., Li Y., Wang X., Fu Y., Qin M., Zhang Y. (2023). In-situ lignin sulfonation for enhancing enzymatic hydrolysis of poplar using mild organic solvent pretreatment. Bioresour. Technol..

[48] Wen J.-L., Sun S.-L., Xue B.-L., Sun R.-C. (2013). Recent Advances in Characterization of Lignin Polymer by Solution-State Nuclear Magnetic Resonance (NMR) Methodology. Materials.

[49] Zhou H., Xu J.Y., Fu Y., Zhang H., Yuan Z., Qin M., Wang Z. (2019). Rapid flow-through fractionation of biomass to preserve labile aryl ether bonds in native lignin. Green Chem..

[50] Nitsos C.K., Choli-Papadopoulou T., Matis K.A., Triantafyllidis K.S. (2016). Optimization of Hydrothermal Pretreatment of Hardwood and Softwood Lignocellulosic Residues for Selective Hemicellulose Recovery and Improved Cellulose Enzymatic Hydrolysis. ACS Sustain. Chem. Eng..

[51] Shao Z., Fu Y., Wang P., Zhang Y., Qin M., Li X., Zhang F. (2020). Modification of the aspen lignin structure during integrated fractionation process of autohydrolysis and formic acid delignification. Int. J. Biol. Macromol..

[52] Li N., Yan K., Rukkijakan T., Liang J., Liu Y., Wang Z., Nie H., Muangmeesri S., Castiella-Ona G., Pan X. (2024). Selective lignin arylation for biomass fractionation and benign bisphenols. Nature.

[53] Geng H., Zhuang L., Li M., Liu H., Caruso F., Hao J., Cui J. (2020). Interfacial Assembly of Metal–Phenolic Networks for Hair Dyeing. ACS Appl. Mater. Interfaces.

[54] Yang Z., Guo W., Yang P., Hu J., Duan G., Liu X., Gu Z., Li Y. (2021). Metal-phenolic network green flame retardants. Polymer.

[55] Liu X., Ni S., Chen X., Li Z., Fu Y., Qin M., Zhang F. (2024). Green fabrication of fabric by ethanol/water solvent-mediated self-assembly of homogeneous lignin for oil–water separation. Green Chem..

[56] Zhang Y., Ni S., Wang X., Zhang W., Lagerquist L., Qin M., Willför S., Xu C., Fatehi P. (2019). Ultrafast adsorption of heavy metal ions onto functionalized lignin-based hybrid magnetic nanoparticles. Chem. Eng. J..

[57] Zhang L., An B., Chen H., Chu J., Ma J., Fan Y., Wang Z. (2022). Botryoidal nanolignin channel stabilized ultrasmall PdNP incorporating with filter membrane for enhanced removal of Cr(VI) via synergetic filtration and catalysis. Sep. Purif. Technol..

[58] Choi J.-H., Park S.-Y., Kim J.-H., Cho S.-M., Jang S.-K., Hong C., Choi I.-G. (2019). Selective deconstruction of hemicellulose and lignin with producing derivatives by sequential pretreatment process for biorefining concept. Bioresour. Technol..

[59] El-Nahhal I.M., Elmanama A.A., Amara N., Qodih F.S., Selmane M., Chehimi M.M. (2018). The efficacy of surfactants in stabilizing coating of nano-structured CuO particles onto the surface of cotton fibers and their antimicrobial activity. Mater. Chem. Phys..

