# Supplementary material for: Direct In Situ Conversion of Both Lignin and Hemicellulose into Single Functional Biopolymers via Biomass Fractionation Process
Source: Polymers (Basel). 2025 Apr 10;17(8):1029. doi: 10.3390/polym17081029 (PMC12030649; doi:10.3390/polym17081029)
Supplement: Supplementary file 1 [file polymers-17-01029-s001.zip › polymers-3538556-supplementary.pdf]

Supporting Materials

Direct In Situ Conversion of Both Lignin and Hemicellulose into Single Functional Biopolymers via Biomass Fractionation Process

Caiyun Liu [1], Shuzhen Ni [1], Zhaojiang Wang [1], Yingjuan Fu [1], Menghua Qin [2] and Yongchao Zhang [1,*]

[1] State Key Laboratory of Green Papermaking and Resource Recycling, Qilu University of Technology, Shandong Academy of Sciences, Jinan 250353, China
[2] School of Chemistry and Chemical Engineering, Qilu Normal University, Jinan 250200, China
* Correspondence: yczhang@qlu.edu.cn

Supplementary Figures

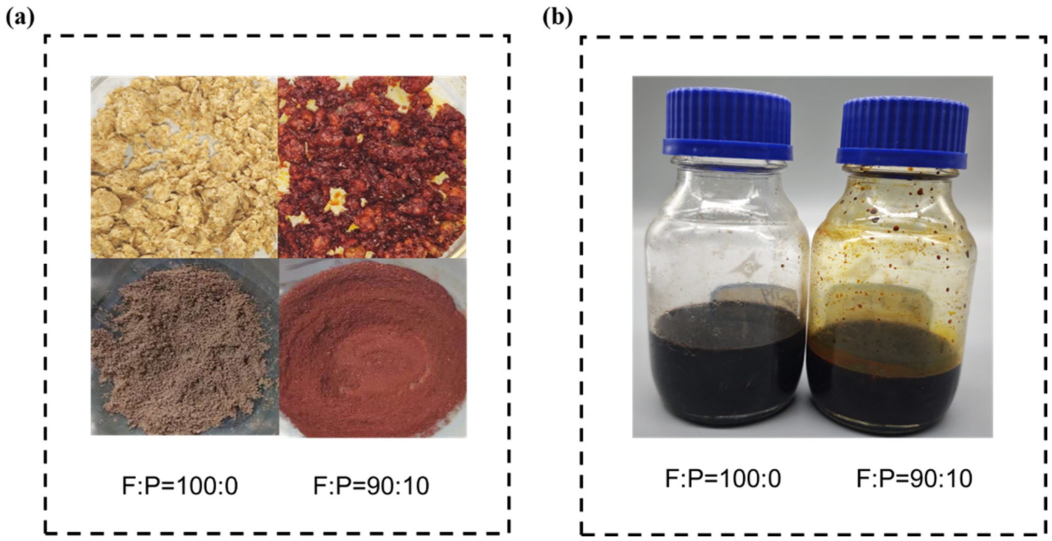

Figure S1. (a) Comparison of the color of residual substrates and lignin, (b) Comparison of the color of the extract.

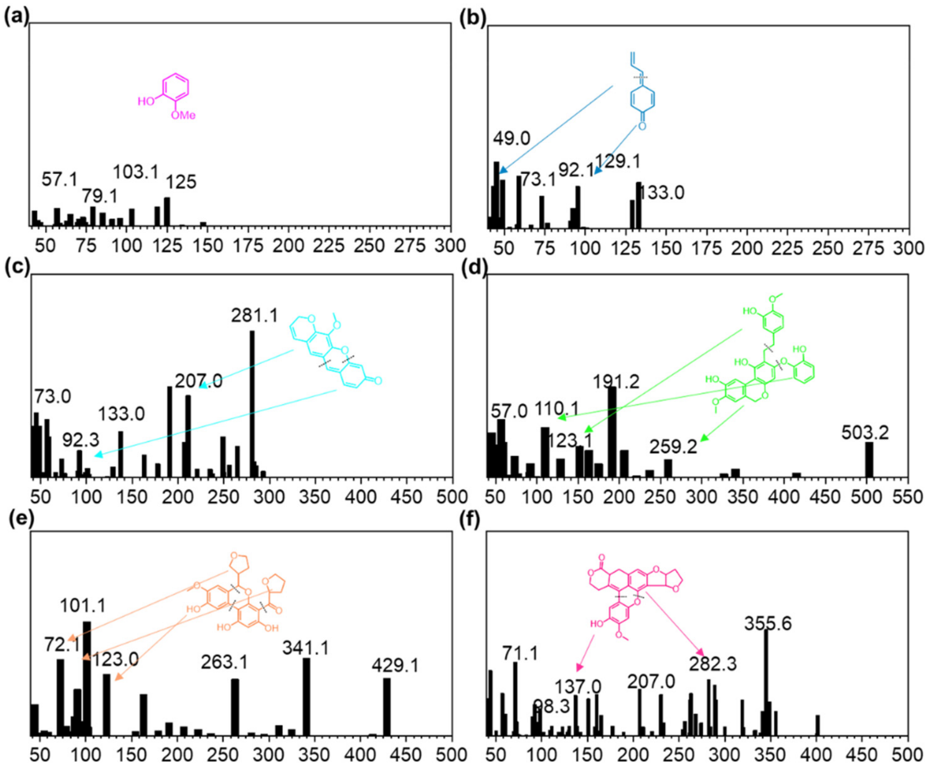

Figure S2. The mass spectrum of purified products separated from GGPL and GGPXL.

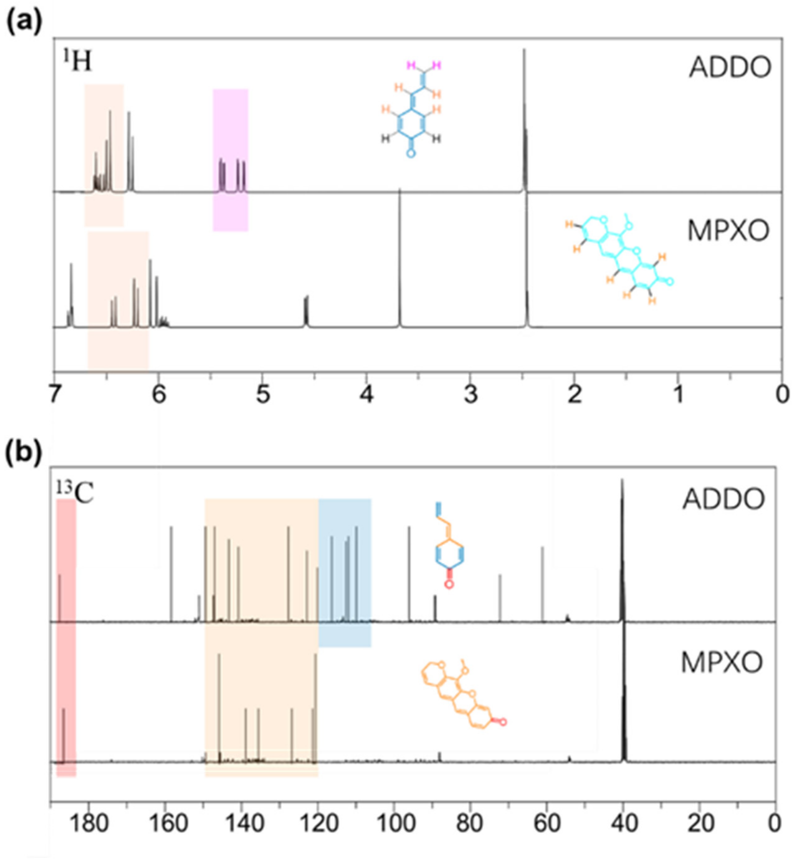

Figure S3. The ^{1}H NMR and ^{13}C NMR spectra for ADDO and MPXO

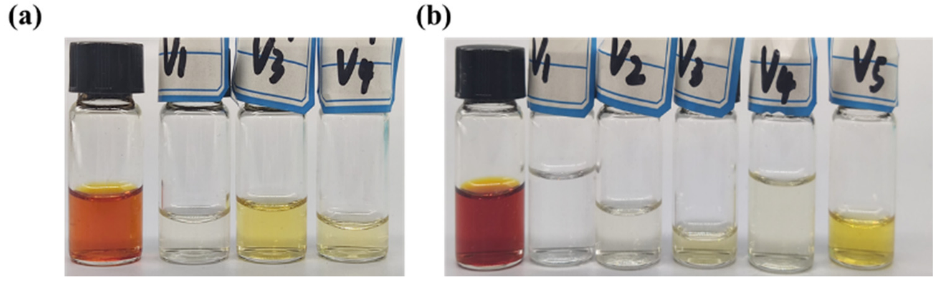

Figure S4. The products were obtained by separation and purification of VGPL (a) and VGPXL (b).
